# Investigation into the Acoustic Properties of Polylactic Acid Sound-Absorbing Panels Manufactured by 3D Printing Technology: The Influence of Nozzle Diameters and Internal Configurations

**DOI:** 10.3390/ma17030580

**Published:** 2024-01-25

**Authors:** Simona Matei, Mihai Alin Pop, Sebastian-Marian Zaharia, Mihaela Coșniță, Cătălin Croitoru, Cosmin Spîrchez, Cristina Cazan

**Affiliations:** 1 Department of Materials Science, Transilvania University of Brasov, 500036 Brasov, Romania; simona.matei@unitbv.ro; 2 Department of Manufacturing Engineering, Transilvania University of Brasov, 500036 Brasov, Romania; zaharia_sebastian@unitbv.ro; 3 Department of Product Design, Mechatronics and Environment, Transilvania University of Brasov, 500036 Brasov, Romania; mihaela.cosnita@unitbv.ro (M.C.); c.vladuta@unitbv.ro (C.C.); 4 Materials Engineering and Welding Department, Transilvania University of Brasov, 500036 Brasov, Romania; c.croitoru@unitbv.ro; 5 Wood Processing and Design Wooden Product Department, Transilvania University of Brasov, 500036 Brasov, Romania; cosmin.spirchez@unitbv.ro

**Keywords:** acoustic properties, 3D printing process, sound-absorbing panels, polylactic acid

## Abstract

Sound-absorbing panels are widely used in the acoustic design of aircraft parts, buildings and vehicles as well as in sound insulation and absorption in areas with heavy traffic. This paper studied the acoustic properties of sound-absorbing panels manufactured with three nozzle diameters (0.4 mm, 0.6 mm and 0.8 mm) by 3D printing from three types of polylactic acid filaments (Grey Tough PLA; Black PLA Pro; Natural PLA) and with six internal configurations with labyrinthine zigzag channels (Z1 and Z2). The absorption coefficient of the sample with the Z2 pattern, a 5.33 mm height, a 0.6 mm nozzle diameter and with Black PLA Pro showed the maximum value (α = 0.93) for the nozzle diameter of 0.6 mm. Next in position were the three samples with the Z1 pattern (4 mm height) made from all three materials used and printed with a nozzle diameter of 0.4 mm with a sound absorption coefficient value (α = 0.91) at 500 Hz. The highest value of the sound transmission loss (56 dB) was found for the sample printed with a nozzle size of 0.8 mm with the Z2 pattern (8 mm height) and with Black PLA Pro. The extruded material, the nozzle diameter and the internal configuration had a significant impact on the acoustic performance of the 3D-printed samples.

## 1. Introduction

Noise pollution is the excessive and uncontrolled presence of unwanted and disturbing sounds in a given environment, which can have negative effects on human health, wildlife and ecosystems. The additive manufacturing of sound-absorbing panels with high acoustic performance is a topical and intensively researched subject, mainly due to the low cost, short manufacturing time and diversity of materials used in these types of 3D printing processes.

Noise is defined as a compound of sounds that affect the psychological and biological state of humans and other organisms which are found in nature. It is one of the main types of pollution, and constant exposure to it can cause different types of health problems such as hearing loss, cardiovascular diseases, sleep disorders, discomfort, etc. [1,2,3,4]. In this regard, sound-absorbing materials made of environmentally friendly materials [5,6,7] or synthetic materials [7,8,9] have been developed. Of these materials, the most important factor influencing their acoustic performance is the porosity [10,11,12,13]. The most common application for porous materials is sound absorption, for example in room acoustics [14,15] or porous linings in aircraft engines or next to porous laminated composite structures for wings or empennage [16]. Porous materials exhibit favorable acoustic properties, especially those with a high absorption coefficient [17].

One method of developing acoustically efficient materials is fused-filament fabrication (FFF), also known as 3D printing. The 3D printing process allows for complex shapes to be manufactured directly from computer-aided design (CAD) templates by successively adding layers of extruded material [18]. For porous materials, 3D printing processes result in samples whose microstructure is known and can therefore be related to the macroscopic homogenized quantities. There are several examples in the literature where the 3D printing process has been used to manufacture sound absorbers. Setaki et al. [19] obtained such sound absorbers on the basis of the destructive interference principle. With this principle, if a peak of a single wave encounters a minimum of another wave, then the amplitude is equal to the difference between the individual amplitudes. Ghaffarivardavagh et al. [20] presented another approach for sound attenuation, namely a structure that reduces sound transmission in pipes while being permeable and can thus be further used in fluid flows using the same principle (destructive interference).

Liu et al. [21] developed multi-layer perforated sound-absorbing panels using 3D printing technology. The acoustic properties of porous media can be estimated using several material designs of different complexity, among which the BIOT model is the most complex [22]. This model estimates the acoustic behavior based on parameters such as the airflow resistivity, sinuosity, porosity, and others. Ring et al. [23] obtained porous and acoustically efficient absorber structures using the material extrusion (MEX) process and demonstrated the potential of these structures.

Boulvert et al. [24] researched the geometrical factors influencing the acoustic properties and reported a numerical optimization procedure of a continuous-gradient porous layer properties to obtain perfect absorption under normal-incidence conditions for 3D-printed samples. Following the study in [24], the best-performing continuous-gradient microstructure, which provides optimal acoustic reflectance and/or transmission, was designed by a nonlinear conjugate-gradient algorithm for 3D-printed cylindrical samples.

In the studies by Gino Iannace [25] and Maria Grazia De Giorgi et al. [26], natural fibers with good sound absorption coefficients were reported that were similar to synthetic porous materials, and upon increasing the sample thickness, the highest values of the sound absorption coefficient moved towards the lowest frequencies with diverse applications, including building restorations for sound isolation.

Carbajo et al. [27] obtained 3D-printed macro-perforated porous polylactic acid filament (PLA) materials using fused deposition modeling (FDM) with a simple filling pattern that provided open porosities ranging from 8% to 39% with pore sizes of at least 0.2 mm. The experimental results showed a high absorption performance for the samples that exhibited macro-perforations. In addition, a comparative study on predictions obtained with the double-porosity theory in conjunction with the Johnson–Champoux–Allard (JCA) approach using macroscopic parameters obtained by an inverse characterization procedure and absorption measurements showed acceptable agreement.

Gliscinska et al. [28] developed sound-absorbing materials from viscose and polylactic-acid-based composites. They found that the presence of the polymer layer on the surface of the composite material improved the sound absorption. In the low-frequency range of sound, the absorption frequency range tended to expand towards lower frequencies as the thickness of the polymer surface layer increased.

The acoustic performances of sound-absorbing panels are characterized by two important properties: the sound absorption coefficient (α) and the sound transmission loss (STL). The sound absorption coefficient (α) is defined as the ratio of the sound energy absorbed by the medium through which the wave passes to the energy of the incident wave. The sound transmission loss (STL) is defined as the ratio of the sound power that enters the sound attenuator to the transmitted sound power.

In the case of porous materials, in addition to the sound absorption characteristics, many researchers have also studied the sound reflection properties [29]. The study in [29] of the natural behavior of infinite uniform layers of a porous material considered the relationship between pressure and velocity, and the results focused on the evaluation of the intrinsic properties of the material based on the acoustic surface impedance of plane waves using two parameters: the energy deviation index and the real reflection angle. Sound reflection is the phenomenon of a sound returning to the medium from which it originates when it meets the separation surface with another medium, which has a different density [30,31].

Zvonicek et al. [32] analyzed the acoustic properties of 3D-printed sound-absorbing panels made of polylactic acid filament (PLA), polyethylene terephthalate modified with glycol (PET-G) and acrylonitrile styrene acrylate (ASA). These researchers observed that the PLA samples demonstrated the best results for the reflection coefficient. Also, from the data analyzed, in terms of the acoustic performance as well as economic constraints, the ideal combination for 3D printing stringed instruments was PET-G material with either a gyroid or grid infill structure printed with a deposition layer height of 0.3 mm or 0.5 mm.

Monkova et al. [33] found that the reflective properties of PLA samples were influenced not only by the type of structure but also by the porosity and thickness of the samples. In a recent study [34], the influence of arbitrarily varying cross-sectional perforations on the acoustic behavior of 3D-printed PLA parts with a divergent–convergent pattern was studied. The results indicated that the sound absorption of perforated panels with a varying cross section was better than that of perforated panels with a uniform cross section for the given frequency range.

The influence of the spherical perforations and their grading on the acoustic characteristics (sound absorption coefficient and sound transmission loss) of a 3D-printed PLA biodegradable material were experimentally analyzed and simulated. The results demonstrated that the sound absorption coefficient of all the functionally graded perforations was higher at low frequencies [35]. Another study [36] researched the acoustic properties of 3D-printed porous polycarbonate material (PPM). The acoustic tests found that with increasing the perforation angle and with the porosity being constant, the sound absorption decreased. Also, the acoustic results indicated that by adjusting the perforation angle and the airgap behind the sample, a high level of sound absorption at low frequencies could be obtained.

Vasina et al. [37] analyzed the different factors (open–porous material structure, the excitation frequency, the sample thickness and the air gap size) that influenced the sound absorption behavior of ABS samples. From the current state of the literature, it can be stated that additive manufacturing processes, particularly FDM, are increasingly being used in studies that analyze the acoustic performance of sound-absorbing panels [36], but there are unexplored research directions that can bring important results in the acoustic field.

In this paper, sound-absorbing panels with different internal configurations made of different types of PLA filaments were designed and manufactured by 3D printing. The obtained samples were acoustically tested by the transfer function method using an acoustic impedance tube. Within this study, the analyzed acoustic properties were as follows: the sound absorption coefficient (α), the sound transmission loss (STL) and the sound reflection coefficient (β).

## 2. Materials and Methods

### 2.1. Design of Acoustic Test Samples

The sample design was carried out using the SolidWorks 2016 software system in accordance with specific acoustic testing standards [38,39,40]. Two sample designs were used for acoustic testing: Z_1_ and Z_2_, as shown in Table 1.

For each design (Z_1_ and Z_2_), samples of three different thicknesses were designed. Thus, for model Z_1_, the samples were 4 mm, 6.4 mm and 8.8 mm thick, respectively, and for design Z_2_, they were 4 mm, 5.33 mm and 8 mm thick, respectively. The samples were 3D-printed with three nozzle diameters (0.4 mm, 0.6 mm and 0.8 mm) and from three types of polylactic acid filaments (Grey Tough PLA; Black PLA Pro; Natural PLA). These sample sizes were in accordance with the mentioned standards [39,40] as well as with the technical characteristics of the impedance tube used in the acoustic tests.

### 2.2. Three-Dimensional Printing of the Samples

Three types of filaments, based on polylactic acid, were used to manufacture the samples for acoustic testing: Grey Tough PLA [41], Black PLA Pro [42] and Natural PLA [43]. The sound-absorbing panels were 3D-printed using a CreatBot DX-3D printer (Henan Suwei Electronic Technology Co., Ltd., Zhengzhou, China). In this study, PLA filament was chosen as the base because it is based on a thermoplastic polyester made from corn starch or sugar cane, which are renewable resources. Admittedly, other advantages of this type of filament were also considered, such as [44,45] its diverse range of filaments and colors, low cost, ease of 3D printing, low 3D printing temperature (180–220 °C), high printing quality and accuracy and low shrinkage. The manufacturing parameters were selected according to each filament type and were controlled via the 3D printing slicing software CreatBot—CreatWare V6.5.2. The specimens were measured after the 3D printing, and a dimensional precision of approx. 0.1 mm was obtained. The most important manufacturing parameters of the 3D-printed sound-absorbing panels with the 3 types of PLA filaments are presented in Table 2.

**Figure 1:**
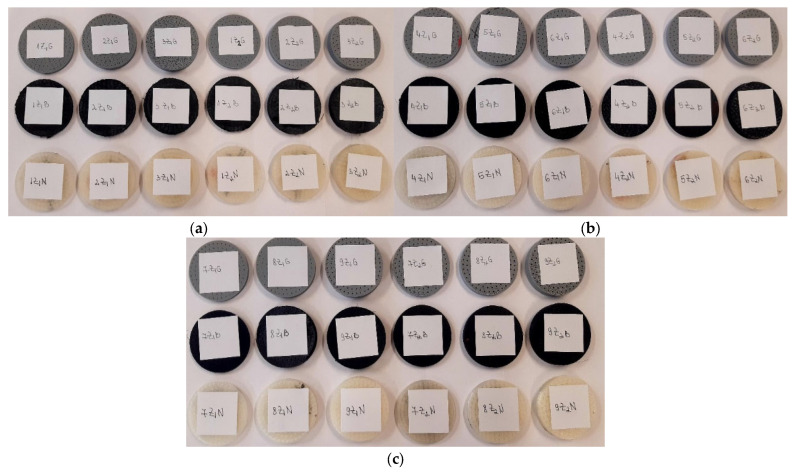
The 3D-printed samples of the three types of materials (Grey Tough PLA; Black PLA Pro; Natural PLA): (**a**) 3D-printed samples with 0.4 mm nozzle; (**b**) 3D-printed samples with 0.6 mm nozzle; (**c**) 3D-printed samples with 0.8 mm nozzle.

The six designs shown in Table 1, with their different thicknesses and internal configurations, were 3D-printed with the three nozzles (0.4 mm; 0.6 mm; 0.8 mm), resulting in 54 samples (Figure 1).

**Figure 2:**
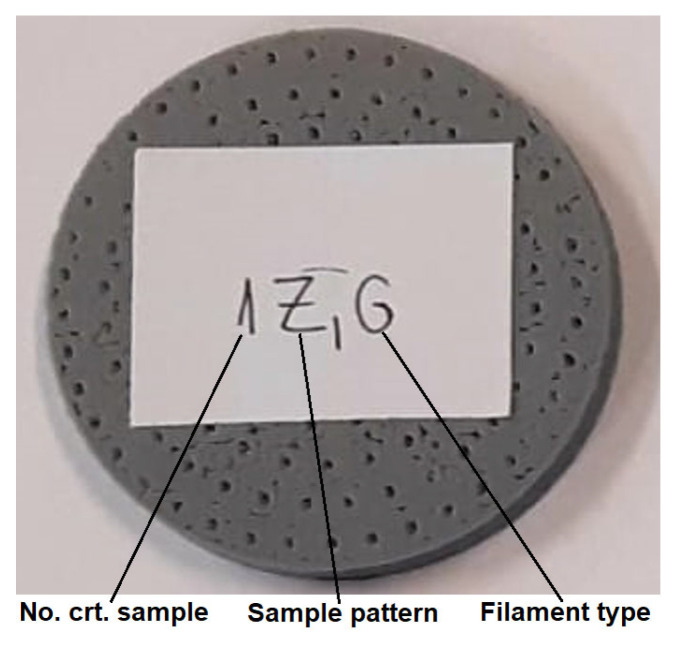
Labelling of the 3D-printed samples.

Depending on the internal configuration of the sample (Z_1_ or Z_2_) and the type of filament (Grey Tough PLA—abbreviated G; Black PLA Pro—abbreviated B; Natural PLA—abbreviated N), the 3D-printed samples were labelled as shown in Figure 2.

### 2.3. Acoustic Analysis of 3D-Printed Samples

**Figure 3:**
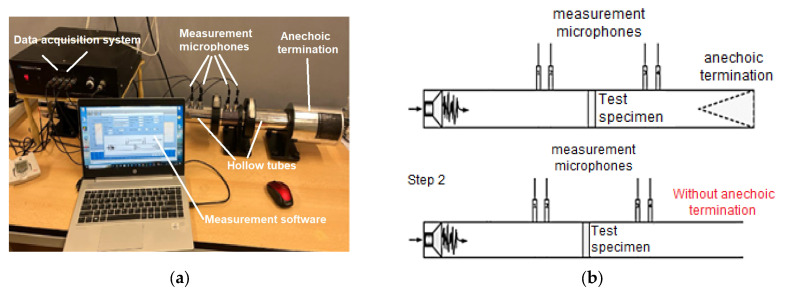
The equipment used for acoustic tests [44]: (**a**) parts of Holmarc HO-ED-A-03 impedance tube; (**b**) description of impedance tube operation.

The acoustic analysis of 3D-printed samples was carried out using a Holmarc HO-ED-A-03 acoustic impedance tube (Holmarc Opto-Mechatronics Ltd., Kochi, India). The impedance system contained an anodized aluminum tube with an inner diameter of 50 mm that could perform the acoustic analysis in the frequency range of 500 Hz–3150 Hz (Figure 3a). The acoustic properties were investigated by the transfer function method according to the current standards [39,40]. The acoustic impedance tube had the following parts: hollow tubes, two pairs of microphones, sample holders, a data acquisition system and measurement software.

In this paper, the frequency dependencies of the sound absorption coefficient (α), the sound transmission loss (STL) and the reflection coefficient (β) of the 3D-printed samples using the transfer function method were investigated. The impedance tube presented two schematic configurations through which the acoustic performance of the 3D-printed sound-absorbing panels could be determined [46]. For the determination of the sound absorption coefficient, the equipment also included an anechoic termination component, and for the sound transmission loss, this anechoic termination part was removed (Figure 3b). When testing each sample, the geometrical parameters of the samples (50 mm), the microphone spacing (30 mm), the temperature and the humidity recorded at each test were entered. For each sample, the height (thickness) was measured, and the sample was then inserted into the impedance tube between the two sets of microphones in a fixed position provided by the device, according to Figure 3b. The device could determine the sound coefficients with samples up to 80 mm.

## 3. Results and Discussion

### 3.1. Influence of Nozzle Diameter on Acoustic Performance of 3D-Printed Samples

A major advantage of 3D printing is that it can produce parts by the changing manufacturing parameters (nozzle diameters, layer height, printing temperature, printing speed, etc.) in a short time. Thus, a total of 54 acoustic tests were carried out for the 3D-printed sound-absorbing panels, with which the following three important parameters were determined: the sound absorption coefficient (α), the sound transmission loss (STL) and the reflection coefficient (β). The values for the 3D-printed samples with nozzle diameters of 0.4 mm, 0.6 mm and 0.8 mm made from the three types of material (Grey Tough PLA; Black PLA Pro; Natural PLA) with the dix configurations (as shown in Table 1) are presented in Table 3, Table 4 and Table 5.

**Figure 4:**
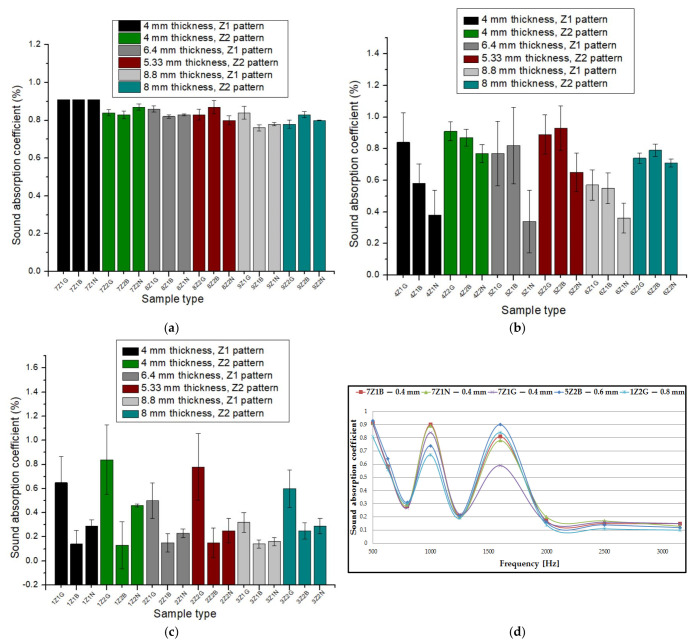
Sound absorption coefficient of 3D-printed sound-absorbing panels: (**a**) samples printed with 0.4 mm nozzle diameter; (**b**) samples printed with 0.6 mm nozzle diameter; (**c**) samples printed with 0.8 mm nozzle diameter; (**d**) variation in the absorption coefficient as a function of frequency.

Figure 4 plots the influence of the sound absorption coefficient (α) as a function of the type of 3D-printed sample. In the case of the sound-absorbing panels printed with a 0.4 mm diameter nozzle (Figure 4a), an almost constant trajectory of the sound absorption coefficient was observed. From Figure 4a, it can be observed that the highest value for the absorption coefficient was found in three types of samples, namely 7Z_1_B, 7Z_1_G and 7Z_1_N. The absorption coefficient value for the three samples was 0.91, which occurred at a frequency of 500 Hz. All the three samples had the same value for the absorption coefficient and had the same sample thickness (4 mm) with the three types of materials analyzed (Grey Tough PLA; Black PLA Pro; Natural PLA). The average of the 18 absorption coefficient values for the nozzle diameter of 0.4 mm was 0.837. For the 3D-printed panels with a 0.6 mm nozzle diameter (Figure 4b), an increase in the absorption coefficient value to 0.93 was found for sample 5Z_2_B, which was recorded at a frequency of 500 Hz. In this case, the average of the 18 absorption coefficient values for the 0.6 mm nozzle diameter was 0.692. In contrast to the panels printed with a 0.4 mm nozzle diameter and a 0.6 mm nozzle diameter, the samples printed with a 0.8 mm nozzle diameter (Figure 4c) showed lower absorption coefficient values. In this case (0.8 mm nozzle diameter), the highest absorption coefficient value was obtained for sample 1Z_2_G (0.84) at a frequency of about 1600 Hz. The average of the 18 absorption coefficient values for the 0.8 mm nozzle diameter was 0.351.

Of all the samples, the highest value for the absorption coefficient (λ = 0.93) was found for sample 5Z_2_B, which means that the noise was very well absorbed. Based on the analysis of the average sound absorption coefficient values for the three nozzle diameters, it can be concluded that the average absorption coefficient values for the 3D-printed samples with a nozzle diameter of 0.4 mm were 17% higher than the average absorption coefficient values of the samples manufactured with a nozzle diameter of 0.6 mm and 58% higher than the average absorption coefficient values of the samples manufactured with a nozzle diameter of 0.8 mm, respectively. Thus, it can be stated that the nozzle diameter had a significant influence on the sound absorption coefficient, and the highest values were shown for a nozzle size of 0.4 mm. This was because the nozzle diameter of 0.4 mm printed with more voids compared to the nozzle sizes of 0.6 mm and 0.8 mm [47], and these small defects (voids between layers of extruded material) are beneficial to better sound absorption [11]. Thus, in acoustic testing, the specific defects of the 3D printing process are an advantage because they create a porosity at the structural level of the sample that leads to higher values of the sound absorption coefficient. The defects that occur improve the passage of sound waves entering the sample, and the air inside the voids can thus move easily, which increases the viscous friction, causing a loss of sound wave energy and, thus, sound is absorbed more efficiently [48]. In Figure 4d, the absorption coefficient curves are plotted for the highest values obtained as a function of the nozzle size, and for the nozzle diameter 0.4 mm, there were three samples with the same absorption coefficient value. As illustrated in Figure 4d, the shape of these curves was similar for all the material types with very small variations, peaking at approximately 500 Hz, 1000 Hz and 1600 Hz. On further analysis of the experimental data, when only the nozzle diameters of 0.4 mm, 0.6 mm and 0.8 mm were varied and the other factors were kept constant (same material, same sample thickness and same internal configuration), it was concluded that about 80% of the maximum absorption coefficient values were obtained with the 0.4 mm nozzle diameter and 20% of the maximum values were obtained for the 0.6 mm nozzle diameter. Thus, it can be stated that another important aspect regarding the FFF process was the correct choice of the deposition layer height (0.2 mm), which is recommended to not exceed the print nozzle diameter (0.4 mm) in order to obtain good mechanical and acoustic performance [49,50,51].

**Figure 5:**
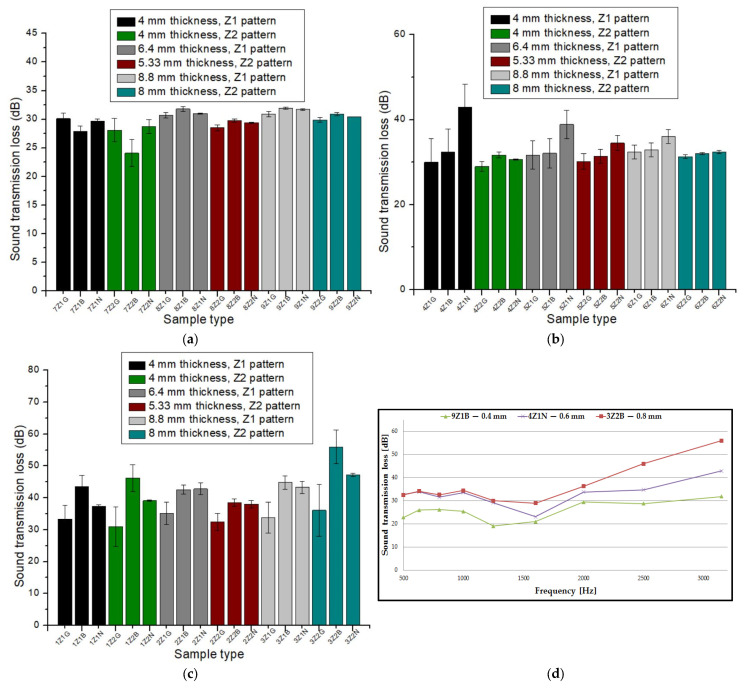
Sound transmission loss of 3D-printed sound-absorbing panels: (**a**) samples printed with 0.4 mm nozzle diameter; (**b**) samples printed with 0.6 mm nozzle diameter; (**c**) samples printed with 0.8 mm nozzle diameter; (**d**) variation in the transmission loss as a function of frequency.

Figure 5 plots the influence of the sound transmission loss (STL) as a function of the 3D-printed sample. In the case of the 3D-printed samples with a nozzle size of 0.4 mm, it can be seen that the highest value for the sound transmission loss was 31.9 dB for sample 9Z_1_B, which was obtained at a frequency of 3150 Hz (Figure 5a). For the 3D-printed samples with a nozzle diameter of 0.6 mm (Figure 5b), an increase in the sound transmission loss up to 42.9 dB was observed for sample 4Z_1_N at the same frequency (3150 Hz). An increase in the sound transmission loss was also observed for the samples printed with a nozzle size of 0.8 mm (Figure 5c). In this case, the highest value was recorded for sample 3Z_2_B (STL = 56 dB). The curves representing the influence of the sound transmission loss as a function of the frequency (Figure 5d) showed similar shapes, with a peak of values for all samples at 3150 Hz.

After analyzing the average sound transmission loss values as a function of the three nozzle diameters, it can be noted that the 3D-printed samples with a nozzle size of 0.6 mm showed a 10% increase in values compared to the average 3D-printed samples with a nozzle size of 0.4 mm, and the 3D-printed samples with a nozzle size of 0.8 mm showed a 34% increase in values compared to the average 3D-printed samples with a nozzle size of 0.4 mm. A conclusion that can be drawn from the analysis of the samples printed with the three nozzle diameters is that the highest value of the sound transmission loss (comparing the same material, the same sample thickness and the same internal configuration) was determined for the nozzle diameter of 0.8 mm (for all cases). This was attributable to the panel, which acts as a barrier preventing sound transmission. This was because the 3D-printed panels with a 0.8 mm diameter nozzle showed fewer defects as compared to the 3D-printed panels with 0.6 mm and 0.4 mm diameter nozzles [47].

**Figure 6:**
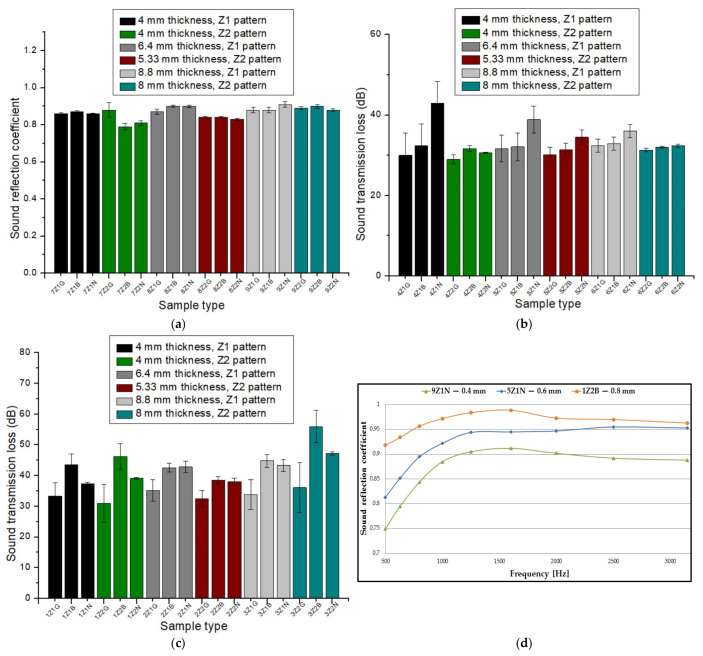
Sound reflection coefficient of 3D-printed sound-absorbing panels: (**a**) samples printed with 0.4 mm nozzle diameter; (**b**) samples printed with 0.6 mm nozzle diameter; (**c**) samples printed with 0.8 mm nozzle diameter; (**d**) variation in the reflection coefficient as a function of frequency.

Figure 6 plots the influence of the reflection coefficient (β) of sound as a function of the sample type. The highest value for the reflection coefficient, in the case of the sound-absorbing panels printed with a 0.4 mm nozzle (Figure 6a), was 0.912 for sample 9Z_1_N. For the samples printed with a nozzle diameter of 0.6 mm (Figure 6b), there was an increase in the reflectivity coefficient, with sample 5Z_1_N showing the highest value of 0.955. The reflectance coefficient further showed an increase to a value of 0.989 for sample 1Z_2_B, which was printed with nozzle size 0.8 mm (Figure 6c).

As can be found in Figure 6d, the three reflection coefficient curves peaked at around 1600 Hz, with high values for all three types of 3D printing nozzle diameters. It can be concluded that as the nozzle diameter increased, the reflection coefficient showed higher values, which was due to the reduced number of defects in the 3D printing with the 0.8 mm nozzle diameter, resulting in a better acoustic reflection of sound waves propagating through the 3D-printed samples.

### 3.2. The Influence of Material Type on the Acoustic Performance of 3D-Printed Samples

The choice of the type of extruded material, in the case of 3D-printed acoustic panels, is one of the important factors in obtaining good acoustic performance. In this sub-chapter, the three types of polylactic acid (PLA), from which the samples were manufactured, were analyzed. This analysis was carried out while keeping the other manufacturing parameters constant (same nozzle diameter, same internal configuration and same sample thickness) and by conducting a horizontal analysis of the materials (Grey Tough PLA; Black PLA Pro; Natural PLA) to determine the influence of the extruded material on the acoustic performance (absorption coefficient, transmission loss and sound reflection coefficient).

As a result of the acoustic tests and a thorough analysis of the three types of materials, the following conclusions can be drawn regarding the sound absorption coefficient:

- For the samples printed with a nozzle diameter of 0.8 mm with the same sample thickness and internal configuration, the Grey Tough PLA material showed the highest values for α (with a minimum of 0.32—sample 3Z_1_—and a maximum of 0.84—sample 1Z_2_G). For the Grey Tough PLA material, these values were 3 times higher as compared to the Black PLA Pro material and double that of the Natural PLA material. An explanation for this high value of α for the Grey Tough PLA printed samples could be attributed to the lower density (1.24 g/cm^3^) of the printed samples that exhibited a higher sound absorption capacity, as demonstrated in other studies [52,53,54].
- In contrast, for the 3D-printed samples with a nozzle diameter of 0.6 mm with the same sample thickness and internal configuration, the absorption coefficient values (with a maximum of 0.91 for Grey Tough PLA—sample 4Z_2_G—and a maximum of 0.93—sample 5Z_2_B for Black PLA Pro) were close for the materials (Grey Tough PLA and Black PLA Pro). For the Natural PLA material, the values were lower compared to the first two and varied for the Z_1_ configuration (α = 0.34–0.38) and were higher for the Z_2_ configuration (α = 0.65–0.77). But on further analysis, it can be stated that the maximum absorption coefficient values (4Z_2_G and 5Z_2_B) were reached for two (Grey Tough PLA and Black PLA Pro) of the three materials.
- For the samples manufactured with a nozzle diameter of 0.4 mm with the same sample thickness and the same internal configuration, the absorption coefficient was close to the maximum for each material type (Grey Tough PLA with α = 0.91—sample 7Z_1_G; Black PLA Pro with α = 0.91—sample 7Z_1_B; Natural PLA with α = 0. 91—sample 7Z_1_N). Therefore, it can be stated that for the different materials (Grey Tough PLA; Black PLA Pro; Natural PLA) and with the following characteristics, the same absorption coefficient results were obtained: nozzle diameter of 0.4 mm, the same sample thickness (4 mm) and the same internal configuration (Z_1_). Thus, it can be concluded that the FFF additive manufacturing process showed high stability in 3D printing with a 0.4 mm diameter nozzle for the three materials analyzed. The nozzle diameter of 0.4 mm provided, in the case of the acoustically tested samples, a balance between the details of the printed parts (fine details on X and Y axes) and the 3D printing time.

In terms of sound transmission loss, which was analyzed considering the three types of materials, the following can be outlined:

- For the 0.8 mm nozzle diameter with the same sample thickness and the same internal configuration, the Black PLA Pro material showed the highest results (minimum 38.5 dB and maximum 56 dB);
- For the 0. 6 mm nozzle diameter with the same sample thickness and the same internal configuration, the Natural PLA material showed the highest results (minimum 32.4 dB and maximum 42.9 dB);
- For the 0.4 mm nozzle diameter with the same sample thickness and the same internal configuration, the Black PLA Pro material showed the highest results (minimum 24.1 dB and maximum 31.9 dB).

Based on the aforementioned results, the sound transmission loss had a maximum value of 56 dB, but these values are very rare, and most values for the STL are between 30 dB and 40 dB as related to PLA material, with a maximum at high frequency (3150 Hz), as observed in other studies [5,34,35,54].

The sound reflection coefficient (β) measures the vertical propagation of sound waves through 3D-printed samples [33] by using the impedance tube method. A summary of the sound absorption coefficient results is presented as follows: the highest values for the 0.8 mm nozzle diameter were obtained with the Black PLA Pro material; for the 0.6 mm diameter nozzle, the highest values of β were attributed to the Natural PLA material; for the 0.4 mm diameter nozzle, the maximum values were very close (exceeding 0.9), and each material had two maximum values.

### 3.3. The Influence of Internal Configuration on the Acoustic Performance of 3D-Printed Samples

The internal configuration of the 3D-printed samples had a significant influence on the acoustic performance [23,34,35]. In this case, the analysis was carried out on the internal configurations of the samples (the two zigzag configurations Z_1_ and Z_2_), and the constant factors were: a sample thickness of 4 mm, the material (Grey Tough PLA; Black PLA Pro; Natural PLA) and the print nozzle diameter (0.4 mm, 0.6 mm and 0.8 mm). The two internal configurations differed in terms of the tilt angle (for Z_1_, it was 31°; for Z_2_, it was 48°) and the internal configuration width (for Z_1_, it was 1.6 mm; for Z_2_, it was 2 mm).

The two internal configurations were labyrinthine with zigzag channels, as used in various studies [55,56,57,58,59], due to the major advantage they confer; they are the most efficient in terms of broad-band sound absorption, and they offer a light weight and compact size. The analyses of the absorption coefficient and sound transmission loss were carried out for the following sample pairs (1Z_1_G–1Z_2_G; 1Z_1_B–1Z_2_B; 1Z_1_N–1Z_2_N; 4Z_1_G–4Z_2_G; 4Z_1_B–4Z_2_B; 4Z_1_N–4Z_2_N; 7Z_1_G–7Z_2_G; 7Z_1_B–7Z_2_B; 7Z_1_N–7Z_2_N). With regard to the absorption coefficient, among the samples analyzed, an internal configuration (Z_1_) was outlined that showed the maximum values, for all types of material, at a nozzle diameter of 0.4 mm. The best absorption coefficient values (α = 0.91) were obtained for the Z_1_ configuration, for a tilt angle of 31° and for a sample thickness of 4 mm. An important factor that has a strong influence on the sound absorption coefficient is the sample thickness [48,60,61], and this was also validated in this paper, where the best results, for all the types of material studied, were obtained at a sample thickness of 4 mm.

In the case of these types of 3D-printed parts, it was also observed in other specialized studies [5,46,62] that the design of the core and the material itself have good sound-absorbing properties up to a frequency of approximately 2000 Hz, after which the absorption coefficient variation has a downward trend with low-intensity variations. The same type of variation in the absorption coefficient was also encountered in the case of all the analyzed samples made by the authors in this study, which were similarly obtained by 3D printing. It should be noted that the Z_2_ configuration also showed high values for the sound absorption coefficient (at a nozzle diameter of 0.4 mm and a sample thickness of 4 mm), with a decrease of between 6–9% as compared to the Z_1_ configuration. In the case of 3D-printed samples with nozzle diameters of 0.6 mm and 0.8 mm, the maximum absorption coefficient values were obtained for the Z_2_ configuration. From these absorption coefficient values, it can be concluded that the tilt angle of the zigzag channels is another important factor in determining the absorption coefficient of 3D-printed samples.

The sound transmission loss for 3D-printed samples represents their ability to provide sound insulation, and the results were opposed to the absorption confinement. Thus, the highest STL results were obtained for the samples printed with a nozzle diameter of 0.6 mm and 0.8 mm for the Z1 configuration, whereas for the samples printed with a nozzle diameter of 0.4 mm, the transmission loss had the highest values for the Z2 configuration as compared to the Z1 configuration.

## 4. Conclusions

The influence of three factors (nozzle diameter, material and internal configuration) of sound-absorbing panels on their acoustic performance (sound absorption coefficient, sound transmission loss and sound reflection coefficient) were determined by the impedance tube method.

The analysis, according to the diameter of the 3D printing nozzle, showed some important aspects regarding the acoustic properties:

- For the nozzle diameter of 0.4 mm, the highest values of the absorption coefficient were obtained (α = 0.76–0.91);
- For the nozzle diameter of 0.6 mm, the highest value of the absorption coefficient (α = 0.93) was obtained for sample 5Z_2_B (5.33 mm thickness, Black PLA Pro filament and Z_2_ internal configuration);
- For the nozzle diameter of 0.8 mm, the lowest values of the absorption coefficient were recorded;
- The average value for α with a nozzle diameter of 0.4 mm was 17% higher than the value of α for the nozzle diameter of 0.6 mm and 58% higher as compared to α for the nozzle diameter of 0.8 mm;
- Based on the analysis of the samples printed with the three nozzle diameters, the highest value of the sound transmission loss (STL = 0.56 dB) was obtained for the nozzle diameter of 0.8 mm;
- The reflection coefficient showed the highest value (β = 0.989) for sample 1Z_2_B, which was printed with a nozzle size of 0.8 mm, and which had the maximum value that corresponded to the lowest absorption coefficient (α = 0.02 at a frequency of 1600 Hz).

The extruded material used in 3D-printed samples also has an important influence on the acoustic performance. Thus, for the absorption coefficient, the following conclusions were drawn: for a nozzle size of 0.8 mm, the Grey Tough PLA filament had the highest values; for a nozzle size of 0.6 mm, the Grey Tough PLA and Black PLA Pro filaments had close values and the Natural PLA filament had lower values; for a nozzle side of 0.4 mm, the closest maximum values of α were obtained for the three material types. The transmission loss recorded usual values for the PLA materials, showing a maximum of 56 dB for a nozzle size of 0.8 mm for the Black PLA Pro material. The reflection coefficient had the highest values for the nozzle size of 0.8 mm and using the Black PLA Pro material.

Another important factor that was investigated in this study was the internal labyrinthine configuration of the 3D-printed samples with zigzag channels. Thus, the two configurations (Z_1_ and Z_2_) had very close values of α for the three materials and for a nozzle size of 0.4 mm. It was noteworthy that the maximum value of the absorption coefficient (α = 0.91) was obtained for the Z_1_ configuration, a tilt angle of 31° and a sample thickness of 4 mm for the three materials analyzed. The sound transmission loss had the opposite values of the sound absorption coefficient, so the samples printed with a nozzle diameter of 0.6 mm and 0.8 mm, for the Z_1_ configuration, had the highest values.

In conclusion, the sound-absorbing panels proposed in this study can be successfully used in various industrial applications (in automotive manufacturing, they can be used in the hood, the spaces next to the engine block, the interior of a car door panel, etc.; they can also be used in aircraft panels, houses and buildings) due to their high acoustic performance, affordable manufacturing method, lightweight internal structure and long lifetime of 3D-printed polylactic acid material.

## Figures and Tables

**Table 1:** Dimensional characteristics of 3D-printed samples.

Section View (Z_1_)	Top View	Section View (Z_2_)
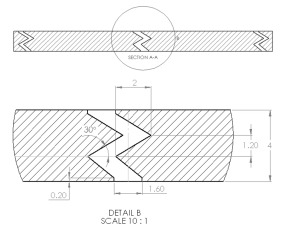	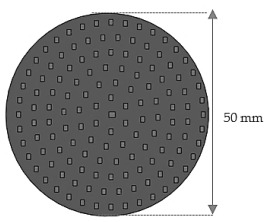	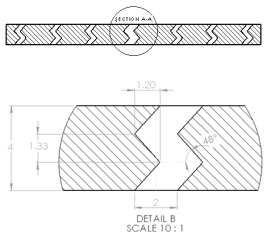
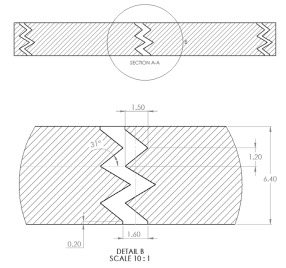		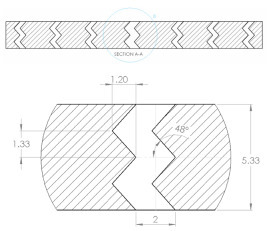
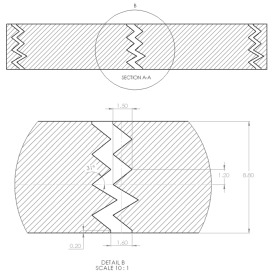		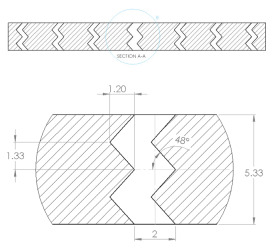

**Table 2:** Filament characteristics and 3D-printed sample parameters [41,42,43].

Parameter	Value		
	Grey Tough PLA	Black PLA Pro	Natural PLA
Filament diameter (mm)	2.85	2.85	2.85
Filament color	Grey	Black	Natural
Printed Part Density (g/cm^3^)	1.22	1.25	1.24
Glass Transition Temperature (°C)	59	63	55–60
Melting Temperature	152	170–180	-
Layer height (mm)	0.2	0.2	0.2
Building plate temperature (°C)	40	40	40
Infill density (%)	60	60	60
Infill pattern	Cubic	Cubic	Cubic
Print speed (mm/s)	40	40	40
Travel speed (mm/s)	120	120	120
Printing temperature (°C)	215	210	210
Top layers	4	4	4
Bottom layers	4	4	4
Nozzle diameter (mm)	0.4/0.6/0.8	0.4/0.6/0.8	0.4/0.6/0.8

**Table 3:** The acoustic test results for the 3D-printed samples (nozzle diameter 0.4 mm).

No.	Filament Type	Acoustic Properties	Nozzle Diameter (0.4 mm)					
			Z_1_ Pattern			Z_2_ Pattern		
			4 mm	6.4 mm	8.8 mm	4 mm	5.33 mm	8 mm
1.	Grey Tough PLA	Sample type	7Z_1_G	8Z_1_G	9Z_1_G	7Z_2_G	8Z_2_G	9Z_2_G
		α	0.91	0.86	0.84	0.84	0.83	0.78
		STL (dB)	30.1	30.7	30.9	28.1	28.5	29.9
		β	0.863	0.877	0.889	0.885	0.847	0.89
2.	Black PLA Pro	Sample type	7Z_1_B	8Z_1_B	9Z_1_B	7Z_2_B	8Z_2_B	9Z_2_B
		α	0.91	0.82	0.76	0.83	0.87	0.83
		STL (dB)	27.9	31.8	31.9	24.1	29.8	30.9
		β	0.879	0.9	0.887	0.793	0.845	0.907
3.	Natural PLA	Sample type	7Z_1_N	8Z_1_N	9Z_1_N	7Z_2_N	8Z_2_N	9Z_2_N
		α	0.91	0.83	0.78	0.87	0.80	0.80
		STL (dB)	29.7	31	31.7	28.7	29.4	30.4
		β	0.868	0.902	0.912	0.81	0.833	0.883

**Table 4:** The acoustic test results for the 3D-printed samples (nozzle diameter 0.6 mm).

No.	Filament Type	Acoustic Properties	Nozzle Diameter (0.6 mm)					
			Z_1_ Pattern			Z_2_ Pattern		
			4 mm	6.4 mm	8.8 mm	4 mm	5.33 mm	8 mm
1.	Grey Tough PLA	Sample type	4Z_1_G	5Z_1_G	6Z_1_G	4Z_2_G	5Z_2_G	6Z_2_G
		α	0.84	0.77	0.57	0.91	0.89	0.74
		STL (dB)	30	31.7	32.4	29	30.2	31.3
		β	0.937	0.918	0.931	0.89	0.899	0.933
2.	Black PLA Pro	Sample type	4Z_1_B	5Z_1_B	6Z_1_B	4Z_2_B	5Z_2_B	6Z_2_B
		α	0.58	0.82	0.55	0.87	0.93	0.79
		STL (dB)	32.4	32.1	32.9	31.7	31.4	32
		β	0.914	0.905	0.937	0.897	0.889	0.873
3.	Natural PLA	Sample type	4Z_1_N	5Z_1_N	6Z_1_N	4Z_2_N	5Z_2_N	6Z_2_N
		α	0.38	0.34	0.36	0.77	0.65	0.71
		STL (dB)	42.9	38.9	36.1	30.6	34.5	32.4
		β	0.942	0.955	0.944	0.893	0.92	0.93

**Table 5:** The acoustic test results for the 3D-printed samples (nozzle diameter 0.8 mm).

No.	Filament Type	Acoustic Properties	Nozzle Diameter (0.8 mm)					
			Z_1_ Pattern			Z_2_ Pattern		
			4 mm	6.4 mm	8.8 mm	4 mm	5.33 mm	8 mm
1.	Grey Tough PLA	Sample type	1Z_1_G	2Z_1_G	3Z_1_G	1Z_2_G	2Z_2_G	3Z_2_G
		α	0.65	0.50	0.32	0.84	0.78	0.60
		STL (dB)	33.4	35.2	33.8	31	32.5	36.1
		β	0.911	0.94	0.957	0.923	0.921	0.93
2.	Black PLA Pro	Sample type	1Z_1_B	2Z_1_B	3Z_1_B	1Z_2_B	2Z_2_B	3Z_2_B
		α	0.14	0.15	0.14	0.13	0.15	0.25
		STL (dB)	43.6	42.6	44.8	46.2	38.5	56
		β	0.983	0.983	0.983	0.989	0.981	0.958
3.	Natural PLA	Sample type	1Z_1_N	2Z_1_N	3Z_1_N	1Z_2_N	2Z_2_N	3Z_2_N
		α	0.29	0.23	0.16	0.46	0.25	0.29
		STL (dB)	37.3	42.8	43.3	39.1	38	47.2
		β	0.957	0.955	0.974	0.927	0.959	0.951

## Data Availability

Data are contained within the article.
